# Clinical Society of Maryland

**Published:** 1892-11

**Authors:** Wm. T. Watson

**Affiliations:** Sec’y; 1519 Broadway, Baltimore, Md.


### CLINICAL SOCIETY OF MARYLAND.

### STATED MEETING HELD JUNE 3, 1892.

The 268th regular meeting of the Society was called to order by the President, Dr. Robert W. Johnson.

DR. HIRAM WOODS related a case of ectropion of both upper lids from disease of the orbital roof, and exhibited the patient.

When the patient, a colored boy, first came under Dr. Woods' care he had had abscesses over the upper eye-lid of each eye, which had ruptured spontaneously, leaving fistulous openings about the middle of each lid, from which pus exuded. With a probe, small areas of denuded bone could be felt about an inch back in each orbit. The patient was put upon tonic treatment, and the sinuses healed. The lids were enormously hypertrophied and the entire edge of each lid was fastened with cicatricial tissue to the edge of the orbit. Dr. Woods operated upon one eye in October, 1891, and upon the other three weeks ago. The edge of each lid was dissected from its position and stitched for the time being to the lower lid. The skin was freely undermined and the horizontal incision was converted into a vertical one. The results were highly satisfactory.

DR. W. B. PLATT read a paper on "Rupture of the Plantaris Tendon;" relating four cases that had occurred in his practice.

DR. CHAMBERS was inclined to doubt the existence of such a thing as rupture of the plantaris tendon. From the attachment and relations it would not be likely to rupture. The pain is usually at a distance from the weakest portion of the tendon and the ecchymosis is more abundant than we would expect to find in a rupture of a tendon. Some good surgeons incline to the idea that these symptoms point to the rupture of a blood vessel. The deep veins may be in a varicose condition.

DR. GEORGE H. ROHE related four cases of puerperal insanity, in which he had removed the uterine appendages, and exhibited to the Society the specimens removed.

Case I.—White woman, 33 years of age. Married at seventeen years of age. This marriage resulted in the birth of one child. In two and a half years she became widowed, and four and a half years later married a second time. In 1882, she gave birth to a second child, and immediately afterward suffered from puerperal mania, which lasted five months. She remained well three years and then again developed insanity and was admitted to the insane asylum with acute mania. When admitted to the hospital she was excited and disposed to fight. She had especial aversion to her husband. She indulged in obscene language. She showed no improvement, but a gradual failure of mental faculties. Suffered from incontinence of urine, and paid no attention to the calls of the rectum. Exhibited great excitement during menstrual period.

Physical examination after coming under Dr. Rohe's care in 1891: Unilateral laceration of cervix up to the vaginal junction and intrapelvic induration on the same side. Perineum ruptured into the rectum.

Abdominal section performed October 6th, 1891, and appendages removed. Clinical conditions present: Right ovary cystic; left ovary cystic and adherent in Douglas' cul-de-sac; thickening and congestion of broad ligament on right side.

After history: Patient recovered fairly well from operation. Had an attack of peritonitis, which yielded promptly to the usual treatment of purgation. The stitches were removed on the seventh day and the wound found perfectly united. December 10th, patient dresses and undresses herself. Seems much interested in looking at books. Appetite good; sleeps well; does not indulge in profane and obscene language as much as formerly. A week later, very much interested in plants and flowers in the wards, and waters them regularly. Appetite good, sleeps well; general behavior very much improved. Present time: Improvement continues. Has written several letters to her husband and to her children, showing decided interest in her family life.

Case II.—White woman, aged 37 years; married 13 years; mother of six children. Admitted to the asylum, May 16,

1890. Insanity developed during the period of lactation. Previous to insanity she was amiable, cheerful and industrious. Her mother had been insane and her father was very intemperate. Had been insane three days when admitted. Had a previous attack ten years before, probably in connection with the birth of a former child, but no exact history. Was subject to hallucinations. Thought nearly every man she met was her brother in disguise. Imagined that she had the power of healing by laying on of her hands. Had a decided tendency to expose her person. Menstrual period irregular. Emaciated, with haggard appearance. Appetite poor; slept poorly; nervous and restless during the day. Put upon a special diet of eggs, milk, beeftea, brandy, etc., but improvement was very slow. The approach of her menstrual periods could be predicted by the alteration in her behavior in the ward.

Physical examination: Bilateral laceration of the cervix; thickening of posterior lip; intra-pelvic inflammatory induration of the left side, sensitive to slight pressure.

Operation November 25, 1891. Left ovary was found adherent. Breaking up of the adhesions occasioned some bleeding. Tube on the left side congested and convoluted.

After history: Recovered well from the operation. Sutures were removed on the seventh day. Note, December 17th: Patient cheerful; appetite good; sitting up in her room, sewing; conversation coherent and has at present no hallucinations, no delusions; simply nervous symptoms such as are present in the majority of cases of induced menopause. At the present time is increasing in flesh and strength; complains less and less of headache and backache and converses entirely rationally. Is much interested in the work about the place, and is ready to go home at any time her husband is prepared to make the proper provision for her.

Case III.—White woman, age 39 years. Married fifteen years. Has had seven children, the last one born four months previous to her admission to the hospital in August, 1887. Before insanity, was amiable and industrious and neat about the household affairs. No insanity was ever in her family. Insanity came on suddenly after the birth of the last child. First symptom was that some one was after her trying to kill her. She used vulgar and obscene language. Tried to kill her mother. Her language in the hospital was of the most obscene character. She would tear her clothes, break the furniture and tear the plastering from the walls. These attacks were intermittent. About six months ago she began to fall off and at the time of the operation was pale and thin.

Physical examination: Deep laceration of cervix on both sides, with eversion of the lips of the cervix and enlargement of the uterus.

Operation December 15, 1891: Uterine appendages removed; small cyst in left broad ligament; one ovary was adherent; uterus somewhat enlarged.

After history: Recovery from operation very good. From being one of the worst patients in behavior, language and general character, she became one that could be kept upon the best ward of the house. She is not well and probably never will be. She has gained in flesh; sews, goes out on the lawn, attends the dances regularly and behaves very well. This patient and the first one will probably never be well, as both are in a condition of somewhat advanced dementia; but they have become better patients.

Case IV.—White woman, aged 28 years. Native of North Carolina and resident of Baltimore City. Admitted in 1891, suffering from mania. Mother of three children. Had an attack of insanity after the birth of the first child and another after the birth of the second child. The third attack came on 12½ months after the birth of her third and last child. The second and third attacks considerably after the births of the respective children. The first attack was a true case of puerperal insanity and probably determined the others. When admitted, was in a state of excitement and indulging in obscene language. Her temperature ran up and her heart grew weak. She was put upon digitalis, eggs and milk every two hours. She gained in strength but her mental symptoms were unimproved.

Physical examination: Deeply lacerated perineum, lacerated cervix and prolapsed ovary.

Operation March 9th. Appendages removed. Great enlargement of ovaries of both sides.

In this case, hereditary taint was denied. Her menstrual periods were regular. While at home she was jealous of her husband's sisters. Was fond of drink, but had not access to much of it. Was indolent and careless. Was fond of talking about sexual matters.

After-history: Three weeks after operation, mental condition good, language to physicians chaste, appetite good. May 8th, 1892, was discharged from the hospital, recovered.

This woman up to the time of the operation used the most profane and obscene language Dr. Rohe had ever heard. When she recovered from the effects of the anæsthetic she burst into tears and asked the doctor's pardon for the ugly language she had used. She never afterward used any obscene or insane language to any one connected with the hospital.

In conclusion, Dr. Rohe said: I believe that in these four cases we have a contribution to the etiology of puerperal insanity. I believe that puerperal insanity is a phase of insanity that is due to absorption of septic matter, and when it is recurrent that it is the result of some reflex irritation due to an inflammatory condition in the pelvis or pelvic organs. All the cases which I have examined show some lesion of the genital canal remaining from parturition. The result of the treatment in these cases show this—that if cases are taken before structural alterations have taken place in the brain, before dementia has come on, that in the large majority of cases restitution of the mental faculties can be accomplished. There is another advantage, I believe, in this radical mode of treatment of this condition; that is, that a woman whose appendages have been removed will never have another attack of puerperal insanity at all events.

DR. WINSLOW: Are these selected cases? Are they all the operations which Dr. Rohe has performed for insane conditions since he had been at Spring Grove?

DR. ROHE: This is a series of cases due to one single cause. I have operated upon fifteen cases. In nearly every case there was some lesion of the pelvic organs. I expect to report all of these cases in the future. I believe that I will be able to report four or five as restored mentally. Nearly all have shown evidences of improvement. They are better patients; they are not so disposed to soil, they can be kept on better wards with quieter patients. This is a decided gain for the management of the hospital.

WM. T. WATSON, M. D. Sec'y.

1519 Broadway, Baltimore, Md.

